# The Role of Non-coding RNAs in Viral Myocarditis

**DOI:** 10.3389/fcimb.2020.00312

**Published:** 2020-07-02

**Authors:** Cong Zhang, Yan Xiong, Lijin Zeng, Zhihua Peng, Zhihao Liu, Hong Zhan, Zhen Yang

**Affiliations:** ^1^Division of Emergency Medicine, Department of Emergency Intensive Care Unit, The First Affiliated Hospital, Sun Yat-sen University, Guangzhou, China; ^2^Department of Cardiology, The First Affiliated Hospital, Sun Yat-sen University, Guangzhou, China; ^3^NHC Key Laboratory on Assisted Circulation (Sun Yat-sen University), Guangzhou, China

**Keywords:** viral myocarditis, non-coding RNA, microRNA, long non-coding RNA, circular RNA

## Abstract

Viral myocarditis (VMC) is a disease characterized as myocardial parenchyma or interstitium inflammation caused by virus infection, especially Coxsackievirus B3 (CVB3) infection, which has no accurate non-invasive examination for diagnosis and specific drugs for treatment. The mechanism of CVB3-induced VMC may be related to direct myocardial damage of virus infection and extensive damage of abnormal immune response after infection. Non-coding RNA (ncRNA) refers to RNA that is not translated into protein and plays a vital role in many biological processes. There is expanding evidence to reveal that ncRNAs regulate the occurrence and development of VMC, which may provide new treatment or diagnosis targets. In this review, we mainly demonstrate an overview of the potential role of ncRNAs in the pathogenesis, diagnosis and treatment of CVB3-induced VMC.

## Introduction

Viral myocarditis (VMC) is a non-ischemic inflammatory disease caused by viral infection and related autoimmune disorders, occupying the vast majority of myocarditis. Epidemiological studies have shown that the incidence of VMC is estimated at 1.0–2.2/million (Vos et al., 2015; Olejniczak et al., 2020). VMC usually presents with chest pain, palpitation and dyspnea, and some patients may progress to heart failure, which has bring huge economic burden on patients and societies (Grun et al., 2012). The gold diagnosis standard of VMC is endomyocardial biopsy (EMB), which has a limited clinical application, so the incidence rate of VMC is likely underestimated. So far, there is no specific blood test has been established to diagnose VMC reliably (Olejniczak et al., 2020). Besides, immunosuppressant, antiviral, and circulatory support are the major treatment methods of VMC, but there still are many patients who have progressed to heart failure after these treatments and thus need heart transplantation (Schultz et al., 2009; Pollack et al., 2015; Tschope et al., 2019). Therefore, the exploration of potentially less traumatic diagnosis methods and new therapeutic targets of VMC is important.

As we all know, lots of viruses can cause VMC, including enterovirus, adenovirus, cytomegalovirus, influenza virus, hepatitis C virus, parvovirus, and other viruses (Fairweather et al., 2012; Lobo et al., 2014; Verdonschot et al., 2016; Minhas et al., 2017; Ntusi, 2017; Spartalis et al., 2017). Coxsackievirus B3 (CVB3) is a single positive strand enterovirus, which is the most common pathogen in VMC etiology (Fairweather et al., 2012). Since the first time using CVB3 to induce myocarditis in mice in 1974 by Woodruff, most of the subsequent experimental models of VMC were induced by CVB3 (Gauntt and Huber, 2003). As the same pattern with many other viruses, CVB3 causes VMC through direct damage to host and indirect injury induced by the abnormal immune response of host immune system (Kindermann et al., 2012; Fung et al., 2016). Therefore, the molecular mechanism research of CVB3 induced VMC model can provide a theoretical basis for the clinical diagnosis and treatment.

Non-coding RNAs (ncRNAs) refer to RNAs that are not translated into protein, including transfer RNA (tRNA), ribosomal RNA (rRNA), small nuclear RNA (snRNA), piwi interacting RNA (piRNA), micro RNA (miRNA), long non-coding RNA (lncRNA), and circular RNA (circRNA) (Panni et al., 2019; Smolle et al., 2019; Zhang W. et al., 2019). ncRNAs were once regarded as transcriptional “garbage.” But recent studies suggested that ncRNAs play an important role in viral infection and host antiviral immunity (Chen et al., 2018). It is generally known that a series of pathophysiological changes after the viral infection is determined by the mutual battle between the virus and the host (Barbu et al., 2020). When the virus is in a dominant position, the host is damaged. On the contrary, the host clears the virus through immune response. Therefore, after viral infection, some ncRNAs may change to help the virus escape from the host's immune system, while the other ncRNAs may change to help the host clear the virus (Nicolas, 2017). Future therapies may focus on helping the host clear viruses or decreasing the viral replication by changing the expression of these important ncRNAs. In addition, considering that some ncRNAs of blood will change significantly in the state of disease and have good stability in peripheral circulation, so they have great potential in the field of VMC diagnosis (Regouc et al., 2020; Santos et al., 2020). In this review, we will mainly describe the potential roles of miRNAs, lncRNAs, and circRNAs in the pathogenesis, diagnosis, and treatment of CVB3-induced viral myocarditis.

## VMC Model Induced by CVB3

Although there is no detailed epidemiological incidence data of CVB3-induced VMC, CVB3 is a leading causative pathogen of VMC (Gauntt and Huber, 2003). Therefore, many teams have studied the formation structure, life cycle, cell infection preference of CVB3 and the CVB3-induced VMC model (Fairweather et al., 2012; Garmaroudi et al., 2015).

### Structure of CVB3

CVB3 virus particle is an icosahedral particle without coating, and its diameter is about 30 nm (Gauntt and Huber, 2003). Capsid protein comprises VP1, VP2, VP3, and VP4. Among these, the first three form the external capsid, while the VP4 located at the inner layer of capsid. The viral genome is a linear single-stranded RNA molecule of about 7.4 kb in size. The genome comprises an open reading frame (ORF) in the middle and an untranslated region on both sides. The ORF encodes 11 proteins (four capsid proteins and 2A, 2B, 2C, 3A, 3B, 3C, 3D) (Garmaroudi et al., 2015). Among them, 2A and 3C are viral proteases, which can induce cell death by cutting the host translation process related proteins such as elF4G and DAP5. Besides, 2B and 2C are involved in RNA synthesis, in which 2B can lead to cardiomyocyte death through largely increasing intracellular calcium level caused by inserting itself into endoplasmic reticulum and Golgi membrane. Moreover, 3A and 3B are vital for viral replication (Peischard et al., 2019). 3D acts as an RNA dependent RNA polymerase, which plays an important role in the synthesis of new viral RNA genome (Garmaroudi et al., 2015).

### Life Cycle of CVB3

CVB3 life cycle mainly includes three critical steps: virus entry, viral translation and replication, virus assembly and release (Freimuth et al., 2008; Knowlton, 2008; Pinkert et al., 2009). DAF and CAR are membrane proteins required for CVB3 to enter the cell, and the entry mode is internalization (Bergelson et al., 1995; Shafren et al., 1995; Bewley et al., 1999; Coyne and Bergelson, 2006). After virus entry, viral genome transcribes, and translates various functional and structural proteins. The viral RNAs are copied by RNA polymerase 3D protein, and then the copied genome is transferred to the structural protein to assemble complete viral particles. Finally, the virus promotes host cell death to facilitate the release of virus particles.

### Tissue Specificity and Gender Preference of CVB3 Infection

CVB3 susceptible cells are mainly distributed in the heart and pancreas, while the lung and kidney are relatively tolerant (Cheung et al., 2005; Harvala et al., 2005). In addition, CVB3 infection has a difference between males and females. Clinically, two-thirds of cases of myocarditis occur in men. It was also found that male mice were more likely to develop CVB3-induced myocarditis than females (Gauntt and Huber, 2003). The sex difference may be related to the different immune responses induced by sex hormones.

### Mechanism of CVB3-Induced Myocarditis

As mentioned before, the pathogenesis of CVB3-induced VMC model includes direct myocardial injury by virus and indirect myocardial injury mediated by the immune system. In the process of direct myocardial injury, the functional proteins 2A and 3C of CVB3 block the translation process of host proteins and hinder the metabolism of host cells by cutting the important proteins elF4G and DAP5 (Chau et al., 2007; Lewis et al., 2008). Besides, the breakdown of the endoplasmic reticulum and Golgi apparatus leads to cell death during the process of virus release (van Kuppeveld et al., 1997). In the process of indirect myocardial injury, the virus promotes the release of inflammatory factors by activating innate immune cells (macrophages, NK cells) and acquired immune cells (Th cells). Moreover, the continuous synthesis of virus products in infected host cells can induce the production of cytokines, inflammatory factors and chemokines, thus stimulating the cardiac infiltration of inflammatory cells. In this process, IL-1α/−5 /−6 /−7 can be massively synthesized by infected cardiomyocytes, while IL-1β/−4 /−6 /−8, TNF α, and TNF β can be massively synthesized and released by inflammatory infiltrated leukocytes (Gauntt and Huber, 2003). Subsequently, a large number of inflammatory factors will further promote myocardial injury.

## The Role of miRNAs in CVB3-Induced VMC

**Figure 1:**
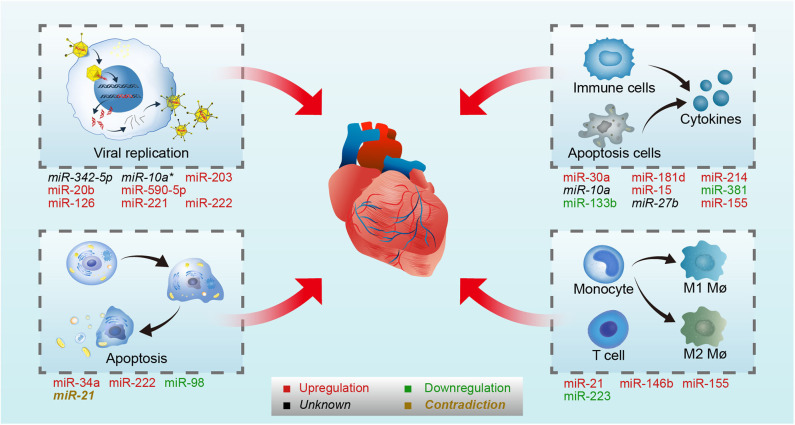
MiRNAs involved in CVB3-induced VMC. CVB3 induced VMC through direct myocardial injury by virus and indirect myocardial injury mediated by the immune system. On one hand, miRNAs regulate viral life cycle (viral replication and host cell apoptosis) by targeting virus genome or host genes in CVB3-induced VMC. On the other hand, miRNAs control immune response by targeting host genes in CVB3-induced VMC. On the upper-left panel, miR-342-5p, /-10a*, /-203, /-20b, /-590-5p, /-126, /-221, and /- 222 involved in viral replication by targeting viral genome or host genes. On the lower-left panel, miR-34a, /-222, /-98, and /-21 involved in host apoptosis. On the upper-right panel, miR-30a, /-181d, /-214, /-10a, /-15, /-381, /-133b, /-27b, and /-155 involved in cytokines production. On the lower-right panel, miR-21, /-146b, /-155, and /-223 involved in immune cells differentiate and activation. Upregulated microRNAs in CVB3 infection are in red, while the downregulated are in green. The microRNA whose expression level were not stated in the original article is in black, and the microRNA whose expression level were contradictory in different studies is in brown. Mø, macrophages.

MiRNAs are a type of endogenous single-stranded RNAs constructed by about 20–25 nucleotides, which can regulate gene expression by binding to the target mRNAs (Dong et al., 2013; Ha and Kim, 2014; Jonas and Izaurralde, 2015; Bracken et al., 2016; Gebert and MacRae, 2019; Treiber et al., 2019). With the deeper and deeper research in recent years, the synthesis mechanism and the function of miRNAs are clarified. The synthesis process of miRNAs contains several steps: transcription, cleavage, export, further cleavage, strand selection, and interaction with mRNA. First, the gene encoding miRNA is transcribed into primary miRNA transcripts which named pri-miRNA with cap structure and polyadenylated tail by RNA polymerase II or RNA polymerase III (Lee et al., 2004). Then, Pri-miRNA is cleaved into about 70–80 nucleotide precursor miRNAs (pre-miRNAs) with hairpin structure by a protein complex consisted of Drosha and its cofactor Pasha (Lee et al., 2003; Denli et al., 2004; Gregory et al., 2004; Han et al., 2004; Landthaler et al., 2004). Subsequently, pre-miRNAs are transported from the nucleus to the cytoplasm by the transporter exportin-5 (Yi et al., 2003; Bohnsack et al., 2004; Kim, 2004; Lund et al., 2004). The pre-miRNAs are cut by Dicer enzyme to form 21 to 25 nt double-stranded miRNA (miRNA duplex) (Grishok et al., 2001; Ketting et al., 2001). After the unwinding of the double-strand, one strand of miRNA duplex was degraded and the other one became of the mature miRNA which then directed into RNA induced silencing complex (RISC) (Gregory et al., 2005; Kobayashi and Tomari, 2016). At last, mature miRNAs regulate target mRNA expression by binding to the target mRNA. When the miRNAs and target mRNAs are completely complementary, miRNAs can lead to degradation of target mRNAs, and the binding site is usually in the coding region of mRNAs (Lai, 2002). By contrast, when they are not completely complementary, miRNAs can inhibit the translation process by binding to the 3′ untranslated region (3′ UTR) of target mRNAs, and finally affect the expression of protein (Bartel, 2004). Recently, it has been found that some miRNAs can also promote gene expression in some cases (Xiao et al., 2017). In the course of VMC, miRNAs have been proved to be differentially expressed, which may regulate the disease development by affecting the life cycle of virus and host immune (Figure 1).

### The Influence of miRNAs on the Life Cycle of CVB3

Viral infection initiates a battle between virus and host. As a type of micro-organism that completely depends on cells to survive, the integrity of life cycle is of great significance for viral infection and infection expansion. Viruses complete their life cycle depends on controlling the survival of host cells. Before the viral load reaches a certain threshold in the cell, the virus inhibits the apoptosis of the host cell, to facilitate viral replication, translation, and assembly. Once viral load reaches the threshold, the virus promotes cell apoptosis to release the amplified virus particles. At the same time, the body is also making efforts to fight against the virus. In the acute phase of virus infection, the body can reduce the infection by inhibiting virus replication. Recent studies have shown that CVB3 can regulate viral replication and host cell apoptosis by affecting miRNAs expression (Chen et al., 2018).

#### MiRNAs on the Replication of CVB3

It has been proven that the replication process is indispensable in the viral life cycle, so inhibiting this process may help to block VMC in the early stages. Undoubtedly, many miRNAs are involved in this process (Table 1; Sodroski et al., 2019; Su et al., 2020). MiRNAs have two patterns to regulate viral replication: one is the direct pattern, and the other is the indirect pattern.

**Table 1:** MiRNAs involved in viral replication.

**MiRNA**	**Target**	**MiRNAs expression**	**Description**	**Process**	**Disease model**	**References**
miR-342-5p	CVB3 2C region	Unknown	miR-342-5p inhibits CVB3 replication by targeting 2C-coding region	Viral replication	*In vitro*: CVB3 (Woodruff strain) infected Hela cells	Wang et al., 2012
miR-10a*	CVB3 3D region	Unknown	miR-10a* promotes CVB3 replication by targeting 3D-coding region	Viral replication	*In vitro*: CVB3 (pKMS1) infected Hela cells	Tong et al., 2013
miR-203	ZFP-148	Upregulation in CVB3 infected Hela cells and A/J mice	PKC/AP-1 activation promotes miR-203 expression miR-203 enhances CVB3 replication by targeting ZFP-148	Viral replication	*In vitro*: CVB3 (Gauntt strain) infected Hela cells; *In vivo*: CVB3 infected A/J mice	Hemida et al., 2013
miR-20b	ZFP-148	Upregulation in CVB3 infected BALB/c mice	miR-20b inhibits ZFP-148 expression and thus enhance CVB3 replication miR-20b promotes cardiomyocytes survival	Viral replication/cell survival	*In vivo*: CVB3 (Nancy strain) infected BALB/c mice	Xu et al., 2017
miR-590-5p	SPRY1	Upregulation in extracellular vesicles released by CVB3 infected HL-1 cells	miR-590-5p enhances CVB3 replication by targeting SPRY1	Viral replication	*In vitro*: CVB3 (pMKS1) infected HL-1 cells	Germano et al., 2019
miR-126	SPRED1, LPR6, WRCH1	Upregulation in CVB3 infected Hela cells	miR-126 enhances CVB3 replication by targeting SPRED1 (ERK1/2 pathway) miR-126 promotes cell death and viral release by targeting LPR6 and WRCH1 (Wnt/β0catenin pathway)	Viral replication/cell death	*In vitro*: CVB3 infected Hela cells	Ye et al., 2013
miR-221/-222	ETS1/2, IRF2, BCL2L11, TOX, BMF, and CXCL12	Upregulation in CVB3 infected C3H and C57Bl6N mice	miR-221/-222 inhibition increases viral replication and immune cell infiltration miR-221/-222 targets ETS1/2, IRF2, BCL2L11, TOX, BMF, and CXCL12 TOX, CXCL12, and IRF2 inhibition suppressed inflammatory response	Viral replication/ inflammation	*In vitro*: CVB3 (Nancy strain) infected neonatal rat cardiomyocytes; *In vivo*: CVB3 (Nancy strain) infected C3H and C57Bl6N mice	Corsten et al., 2015

*ZFP-148, zinc finger protein-148; SPRY1, sprout 1; SPRED1, sprouty-related EVH1 domain-containing protein 1; LRP6, lipoprotein receptor-related protein 6; WRCH1, Wnt responsive Cdc42 homolog 1; IRF2, interferon regulatory factor 2; BCL2L11, Bcl-2-like protein 11; TOX, Thymocyte selection-associated high-mobility group box; BMF, BCL-2-modifying factor; CXCL12, CXC chemokine ligand 12*.

The direct pattern means that miRNAs directly target the viral genome sequence to inhibit or promote viral replication. Wang et al. have found that miR-342-5p may target the 2C-coding region of the viral genome by bioinformatic analysis. Further experiments confirmed that miR-342-5p can directly inhibit viral replication by targeting the 2C-coding region of CVB3 (Wang et al., 2012). Unlike with most negatively regulated traditional miRNAs, some miRNAs can promote mRNA expression. Wang's group found that miR-10a^*^ directly promotes the viral biosynthesis by targeting 3D-coding region (nt6818-nt6941) of CVB3. Additionally, miR-10a^*^ is abundant in heart of Balb/c mice which indicated that miR-10a^*^ may affect VMC via inducing CVB3 replication (Tong et al., 2013). These findings implied that miRNAs might be a useful treatment by directly limiting viral replication.

The indirect pattern means that miRNAs indirectly control viral replication by targeting the host genome. Many genes, such as zinc finger protein (ZFP)-148, sprout 1 (SPRY1), sprouty-related EVH1 domain-containing protein 1 (SPRED1), and E-twenty six 1/2 (ETS1/2), regulate replication in viral infection. Studies have shown that the upregulation miRNAs, such as miR-203 and miR-20b, targeted ZFP-148 to promote viral replication and cell survival in CVB3 induced VMC (Hemida et al., 2013; Xu et al., 2017). The miRNAs secreted by cells may also act as complex regulators on viral infection. Germano et al. showed that the levels of miR-590-5p increased in vesicles shed from infected cells, and this miRNA prolong viral replication and enhance cell survival via targeting SPRY1 (Germano et al., 2019). Besides, extracellular signal-regulated kinase 1/2 (ERK1/2) and Wnt/β-catenin pathways were involved in the VMC. CVB3 infection promotes ERK1/2 phosphorylation, which activated ETS transcriptional protein activity, causing miR-126 expression upregulation. Then miR-126 blocked the target gene SPRED1, lipoprotein receptor-related protein 6 (LRP6) and Wnt responsive Cdc42 homolog 1 (WRCH1) to promote the crosstalk with ERK1/2 and Wnt/β-catenin pathways. Finally, the ERK1/2 pathway accelerated the viral replication, and the Wnt/β-catenin pathway promoted virus-mediated cell death and viral release (Ye et al., 2013). Therefore, CVB3 infection can induce the upregulation of miR-126 and promote virus replication and release by regulating ERK1/2 and Wnt/β-catenin pathways.

The body did not await its doom in CVB3 infection. Corsten et al. found that CVB3 infection promoted the miR-221/-222 expression in cardiac tissue. Further study demonstrated that miR-221/-222 could inhibit ETS1/2, interferon regulatory factor 2 (IRF2), Bcl-2-like protein 11 (BCL2L11), Thymocyte selection-associated high-mobility group box (TOX), BCL-2-modifying factor (BMF), and CXC chemokine ligand 12 (CXCL12) As we all know, those target genes are important in viral replication and inflammation (Welsh, 1986; Bosselut et al., 1990; Sieweke et al., 1998; Posada et al., 2000; Kühl et al., 2003; Zhan et al., 2005; Russell and Garrett-Sinha, 2010; Cheng et al., 2011; Johansson et al., 2011; Tanaka-Nakanishi et al., 2014; Okuyama et al., 2018). The *in-vitro* study confirmed that miR-221/-222 inhibited inflammation through those IRF2, CXCL12 and TOX in CVB3 infection (Corsten et al., 2015). This study indicated that the body tried to eliminate the virus and protect heart by increasing the expression of miR-221/-222 reactively during CVB3 infection.

All in all, these studies confirmed that the expression of miR-342-5p, miR-10a ^*^, miR-203, miR-20b, miR-590-5p, and miR-126 could promote viral replication, and the body could also reactively promote the expression of miR-221/-222 to reduce viral replication. In the future, we can reduce CVB3 replication by targeting these miRNAs in VMC treatment.

#### The Effect of miRNAs on Host Cell Apoptosis During CVB3 Infection

The virus completes its life cycle by controlling apoptosis (Kvansakul, 2017). In the early stage of viral infection, the virus promotes cell survival by inhibiting cell apoptosis to ensure sufficient amplification. In the later stage of infection, the virus promotes cell apoptosis to facilitate release and spread. MiR-34a, miR-222, miR-98, and miR-21 play an important role in cell apoptosis (Zhou et al., 2017; Tong et al., 2019; Zheng et al., 2019; Shen et al., 2020). Therefore, many researchers focus on the role of miRNAs in virus-induced cell apoptosis. Jiang et al. found that inhibition of miR-34a expression largely decreased the level change of apoptosis-related protein, including Bax and Bcl-2 (Jiang et al., 2019). Sirtuin 1 (SIRT1) was a validated target gene of miR-34a (Castro et al., 2013; Yang et al., 2015; Carloni et al., 2016). Then the researchers confirmed miR-34a act as a pro-apoptotic molecule in VMC via targeting the SIRT1-p53 signaling pathway (Jiang et al., 2019). In contrast, miR-222, miR-98, and miR-21 may act as anti-apoptotic factors in VMC (He et al., 2013; Zhang B. Y. et al., 2016; Zhang X. et al., 2019). Zhang et al. found that the expression of adenosine deaminase, RNA-specific (ADAR1) and miR-222 was increased in CVB3 induced VMC model, and the study *in vitro* showed that ADAR1 combined Dicer increased cell viability by inducing miR-222 synthesis which decreased the confirmed target phosphatase and tensin homolog (PTEN) expression (Zhang X. et al., 2019). Previous studies have proven that PTEN is an apoptosis protein (Dupont et al., 2002; Lin et al., 2007; Cheng et al., 2009). Hence, ADAR1-Dicer Complex may inhibit cell apoptosis by miR-222 in CVB3 induced VMC. Additionally, MiR-98 decreased cell apoptosis by targeting the FAS/FASL gene (Zhang B. Y. et al., 2016), and miR-21 reduced cell apoptosis by targeting programmed cell death 4 (PDCD4) (He et al., 2013), a well-known apoptosis gene (Gaur et al., 2011; Stagakis et al., 2011; Junker et al., 2015). Furthermore, another study confirmed that miR-21 reduced cell apoptosis by targeting mitogen-activated protein kinase kinase 3 (MAP2K3) (He et al., 2019). Interestingly, although both studies showed that miR-21 has an anti-apoptosis effect, the expression trend of miR-21 was not consistent after CVB3 infection in different studies (Table 2). The conflicting results may be related to the different viral strains used and different sample acquisition time in two studies. All in all, these studies show that the virus can complete its life cycle by influencing the level of miRNAs to regulate host cell apoptosis. In the future, we can target these miRNAs to destroy the life cycle of the virus to reduce body injury.

**Table 2:** MiRNAs involved in cell apoptosis.

**MiRNA**	**Target**	**MiRNAs expression**	**Description**	**Process**	**Disease model**	**References**
miR-34a	SIRT1	Upregulation in CVB3 infected neonatal rat cardiomyocytes	miR-34a promotes cell apoptosis via SIRT/p53pathway	Apoptosis	*In vitro*: CVB3 (Nancy strain) infected neonatal rat cardiomyocytes	Jiang et al., 2019
miR-222	PTEN	Upregulation in CVB3 infected H9C2 cells, primary cardiac cells, and BALB/c mice	ADAR1 combined Dicer induced miR-222 synthesis miR-222 decreases PTEN expression ADAR1 increases cell viability via regulating PTEN expression	Apoptosis	*In vitro*: CVB3 infected H9C2 and primary cardiac cells; *In vivo*: CVB3 infected BALB/c mice	Zhang X. et al., 2019
miR-98	FAS/FASL	Downregulation in blood of VMC patients	miR-98 decreases and FAS/FASL increased in VMC patients miR-98 inhibits apoptosis via targeting FAS/FASL	Apoptosis	VMC patients	Zhang B. Y. et al., 2016
miR-21	PDCD4	Downregulation in CVB3 infected BALB/c mice	miR-21 inhibits cell apoptosis via PDCD4	Apoptosis	*In vivo*: CVB3 (Nancy strain) infected BALB/c mice	He et al., 2013
miR-21	MAP2K3	Upregulation in CVB3 infected BALB/c mice and Hela cells	miR-21 inhibits cell apoptosis via MAP2K3/p38 MAPK pathway miR-21 does not affect CVB3 replication	Apoptosis	*In vitro*: pCVB3M strain infected Hela cells; *In vivo*: pCVB3M strain infected BALB/c mice	He et al., 2019

*SIRT1, Sirtuin 1; ADAR1, adenosine deaminase, RNA-specific; PTEN, phosphatase and tensin homolog; PDCD4, programmed cell death 4; MAP2K3, mitogen-activated protein kinase kinase 3*.

### MiRNAs on Host Immune and Inflammatory Response

The CVB3-induced immune response is very complex, including the activation of innate immunity and acquired immunity. In the early stage of CVB3 infection, innate immune-related cells (macrophages and NK cells) infiltrate the heart, producing chemokines and Interferon (IFNs). CVB3 infection can promote the polarization of M2 macrophages toward M1 macrophages, which increases the inflammation (Zhang Y. et al., 2016; Gou et al., 2018). Besides, the 2A protein of CVB3 inhibits the production of IFNs by targeting TLR3 and MDA5/RIF-I pathways, which are vital pathways in host antiviral immunity (Lind et al., 2016). In the subsequent stage, acquired immune-related cells (Th1, Th2, and Th17 cells) are activated to produce cytokines and inflammatory factors (Gauntt and Huber, 2003). Th1 cells mainly produce IFN-γ, which can promote inflammation. Moreover, Th2 cells mainly produce IL-10, which has an anti-inflammatory effect. Furthermore, Th17 cells mainly produce IL-17, which plays a key role in promoting CVB3 induced infection (Garmaroudi et al., 2015). In recent years, researchers also focus on the field that miRNAs participate in the inflammation process by activating innate and acquired immune responses during CVB3 infection (Table 3).

**Table 3:** MiRNAs involved in immune and inflammation.

**MiRNA**	**Target**	**MiRNAs expression**	**Description**	**Process**	**Disease model**	**References**
miR-21, miR-146b	ROR-γt	Upregulation in CVB3 infected BALB/c mice	Inhibition of miR-21 or miR-146b decreased the proportion of Th17 cells via targeting ROR-γt miR-21 or miR-146b decreased IL-17, IL-6, and TGF-β levels	Th17 cells differentiation	*In vivo*: CVB3 (Nancy strain) infected BALB/c mice	Liu et al., 2013
miR-155	PU.1	Upregulation in CVB3 infected C3H mice, C57Bl6 mice and VMC patients	Inhibition of miR-155 relieved cardiac injury by inhibiting macrophage infiltration and T cell activation PU.1 is the direct target gene of miR-155 Inhibition of miR-155 does not affect viral replication	Macrophage infiltration and T cell activation	*In vivo*: CVB3 infected C3H mice and C57Bl6 mice; VMC patients	Corsten M. et al., 2012
miR-155	–	Upregulation in CVB3 infected miR-155 knock out C57Bl6 mice	Silencing miR-155 suppresses M1 macrophages polarization, and promotes macrophages toward to M2 phenotype	Macrophages polarization	*In vivo*: CVB3 (Nancy strain) infected C57Bl6 mice	Zhang Y. et al., 2016
miR-155	RelA	Upregulation in myocardial tissue of VMC patients	miR-155 overexpression decreases inflammatory factor to reduce cardiac injury by targeting RelA (NF-κB pathway)	NF-κB inflammatory pathway	*In vivo*: CVB3 (Nancy strain) infected BALB/c mice; VMC patients	Bao and Lin, 2014
miR-223	PKNOX1	Downregulation in CVB3 infected BALB/c mice	miR-223 overexpression suppresses M1 macrophages polarization, and promotes macrophages toward to M2 phenotype via targeting PKNOX1	Macrophages polarization	*In vivo*: CVB3 (Nancy strain) infected BALB/c mice	Gou et al., 2018
miR-30a, miR-181d	SOCS3	Upregulation in the blood of VMC patients and CVB3 infected Hela cells	miR-30a and miR-181d enhanced the level of IL-6 by targeting SOCS-3	Proinflammatory factor	*In vitro*: CVB3 (Nancy strain) infected Hela cells; VMC patients	Fan et al., 2019
miR-214	ITCH	Upregulation in the right ventricular septal specimens of VMC	miR-214 increases TNF-α, IL-1β, MCP-1, and IL-6 by targeting ITCH (NF-κB pathway)	Proinflammatory factor	*In vitro*: CVB3 infected Hela cells; VMC patients	Chen et al., 2015
miR-10a	ITCH	Unknown	Allele A of rs3809783 in pri-miR-10a coding region in the VMC population was related to VMC occurrence miR-10a promoted IL-6 expression by targeting ITCH (NF-κB pathway)	Proinflammatory factor	*In vitro*: CVB3 infected Hela cells; VMC patients	Liao et al., 2015
miR-15	NLRX-1	Upregulation in CVB3 infected H9C2 cells	miR-15 promotes the expression of IL-1β, IL-6, and IL-18 by targeting NLRX-1 to activate NLRP3 inflammasomes miR-15 inhibition suppresses CVB3-induced cell apoptosis	Proinflammatory factor	*In vitro*: CVB3 (Nancy strain) infected H9C2 cells;	Tong et al., 2020
miR-381	COX-2	Downregulation in the blood of children with VMC and CVB3 infected BALB/c mice	miR-381 decreases myocardial injury via targeting COX-2	Anti-inflammatory factor	*In vivo*: CVB3 infected BALB/c mice; VMC children	Zhang Y. et al., 2018
miR-133b	Rab27B	Downregulation in the blood of VMC patients	miR-133b reduced IL-6 and TNF-α by directly targeting Rab27B	Anti-inflammatory factor	*In vitro*: CVB3 infected cardiomyocytes; VMC patients	Zhang et al., 2017
miR-27b	MCP1	Unknown	miR-27b inhibited the level of MCP1 in IL-17 treated H9C2 cells	Anti-inflammatory factor	*In vitro*: IL-17 treated H9C2 cells	Huang et al., 2016

*ROR-γt, retinoid-related orphan receptor gamma-t; PU.1, purine-rich box 1; RelA, v-rel avian reticuloendotheliosis viral oncogene homolog A; PKNOX1, PBX/knotted 1 homeobox 1; SOCS3, suppressor of cytokine signaling 3; ITCH, itchy E3 ubiquitin-protein ligase; NLRX-1, NLR family member X1; COX-2, cytochrome c oxidase subunit II; Rab27B, RAB27B, member RAS oncogene family; MCP-1, macrophage chemo-attractant protein-1; TNF-α, tumor necrosis factor-alpha; IL, interleukin*.

#### MiRNAs on T Cells and Macrophages During CVB3 Infection

Monocytes-macrophages and T cells are representative cells in innate and adaptive immune in VMC, respectively (Gauntt and Huber, 2003). Monocytes differentiate into macrophages after they reach the tissues, among which M1 macrophages have the effect of promoting inflammation, while M2 macrophages have the effect of anti-inflammation (Huang et al., 2018a). Th cells (mainly including Th1, Th2, and Th17 cells) also play a role in CVB3 infection (Garmaroudi et al., 2015). Previous studies have shown that IFN-γ knockout mice are free from VMC after CVB3 treatment, while IFN-γ overexpression is susceptible (Huber and Sartini, 2005). Therefore, Th1 cells can secrete IFN-γ to promote the development of VMC. Besides, IL-10 is a vital anti-inflammatory factor, and Th2 cells mainly secrete IL-10 to act as an anti-inflammatory role. As we all know, Th17 cells can secrete IL-17. Previous studies have found that inhibition of IL-17 by neutralizing antibodies can reduce viral replication and myocardial damage in myocarditis, suggesting that Th17 cells are important pro-inflammatory cells (Fan et al., 2011). Proinflammatory factors, such as miR-21 and miR-146b, have been identified that were increased in VMC. Inhibition of miR-21 or miR-146b decreased Th17 cells and relieved myocardial injury by targeting retinoid-related orphan receptor gamma-t (ROR-γt) (Liu et al., 2013). Moreover, miR-155 also acts as a pro-inflammatory factor in some cases and it was upregulated in CVB3 induced VMC model and human myocarditis specimen (Corsten M.F. et al., 2012; Zhang Y. et al., 2016). A research conducted by Corsten et al. illustrated that inhibition of miR-155 relieved cardiac injury by inhibiting macrophage infiltration and T cell activation, and the direct target of miR-155, purine-rich box 1 (PU.1), may function in this process (Corsten M.F. et al., 2012). A further study directed by Zhang et al. showed that inhibition of miR-155 increased M2 macrophages and decreased M1 macrophages to attenuate cardiac inflammatory (Zhang Y. et al., 2016). However, Bao et al. found that miR-155 acts as an anti-inflammatory factor to reduce cardiac injury by targeting v-rel avian reticuloendotheliosis viral oncogene homolog A (RelA) (Bao and Lin, 2014), a vital component of nuclear factor kappa-B (NF-κB) inflammatory pathways (Gasparini et al., 2014; Mukherjee et al., 2015; Kabacaoglu et al., 2019). These contradictory results may be due to the use of different models. Moreover, miR-223, which also acts as an anti-inflammatory factor, has been verified that was decreased in VMC. Forced expression of miR-223 activated M1 macrophages toward M2 anti-inflammatory phenotype through targeting PBX/knotted 1 homeobox 1 (PKNOX1), and thus relieving myocardial inflammation (Gou et al., 2018). Therefore, CVB3 infection can promote immune cells-mediated inflammation by regulating the expression of miRNAs. In the future, we can reduce the degree of myocardial inflammation by targeting these miRNAs.

#### MiRNAs on Cytokines Expression After CVB3 Infection

CVB3-induced cardiomyocytes apoptosis and activated immune cells release many cytokines, such as Interferons (IFNs), tumor necrosis factor-alpha (TNF-α), Interleukin-1 beta (IL-1β), Interleukin-6 (IL-6), Interleukin-10 (IL-10), Interleukin-12 (IL-12) (Kanda et al., 1996; Miyamoto et al., 2001; Matsumori et al., 2004; Lindner et al., 2014; Wei et al., 2019; Yang et al., 2019). Some of these cytokines (Type I IFNs, Type II IFNs, and IL-10, etc.) are anti-inflammatory factors while others (TNF-a, IL-6, and IL-12, etc.) are proinflammatory factors (Corsten M. et al., 2012). MiRNAs also regulated the expression of cytokines during CVB3 infection. An investigation conducted by Fan et al. found that miR-30a and miR-181d increased in CVB3 induced VMC mice model. Further research confirmed that those miRNAs enhanced the level of IL-6 by targeting SOCS-3 (Fan et al., 2019). Analogously, miR-214, increased in VMC, enhanced the level of IL-1β, IL-6, macrophage chemo-attractant protein (MCP)-1, and TNF-α by targeting itchy E3 ubiquitin-protein ligase (ITCH) (Chen et al., 2015), an NF-κB pathway suppressor (Chen et al., 2001; Shembade et al., 2008; Perez et al., 2015). ITCH is also the direct target of miR-10b. Liao et al. found that allele A of rs3809783 in pri-miR-10a coding region in the VMC patients was related to VMC occurrence, and this mutation site reduced mature miR-10a expression. Further research identified that miR-10a promoted IL-6 expression by targeting ITCH (Liao et al., 2015). MiRNAs may promote the development of inflammation through the activation of inflammasomes. Tong et al. found that miR-15 was up-regulated after CVB3 infection. This up-regulated miRNA promotes the expression of IL-1β, IL-6, and IL-18 by targeting NLR family member X1 (NLRX-1) to activate NLRP3 inflammasomes (Tong et al., 2020). On the contrary, miR-381, miR-133b, and miR-27b act as anti-inflammatory factors. MiR-381 relieved myocardial injury by targeting cytochrome c oxidase subunit II (COX-2) (Zhang Y. et al., 2018), miR-133b reduced IL-6 and TNF-α by directly targeting RAB27B, member RAS oncogene family (Rab27B) (Zhang et al., 2017), and miR-27b inhibited the level of MCP1 in IL-17 treated H9C2 cells (Huang et al., 2016). These results indicate that CVB3-induced inflammation was regulated by miRNAs targeted inflammatory signaling pathways.

### Potentiality of miRNAs in the Treatment of CVB3-Induced VMC

As mentioned above, after CVB3 infection, miRNAs function by influencing the viral life cycle and immune response. Therefore, miRNAs may be potential therapeutic targets for VMC. For example, miRNAs such as miR-342-5p, miR-10a ^*^, miR-203, miR-20b, miR-590-5p, and miR-126 can directly or indirectly affect virus replication. We can design inhibitors or analogs of these miRNAs to inhibit CVB3 replication, thereby reducing the viral load in infected host cells. In addition, miRNAs such as miR-21, miR-146b, miR-233, miR-155, miR-27b, miR-30a, miR-181d, miR-214, miR-10a, miR-381, and miR-133b can regulate the CVB3-induced inflammatory response by affecting the differentiation of immune cells or the release of cytokines, so we can design inhibitors or analogs of miRNAs to reduce myocardial inflammation and damage. Clinical trials of miRNAs based therapeutics have been used in other diseases, so it may become a reliable treatment for VMC in the future (Beg et al., 2017).

Although miRNAs-based therapeutics might be a promising treatment in VMC, there are still some problems. First, finding the method to help the miRNAs targeted transport to the heart is critical. MiRNAs can reach all organs of the body through blood circulation, which may cause other organs injury under miRNAs untargeted transporting. Second, one miRNA can target multiple genes at the same time, and one gene may be controlled by multiple miRNAs. MiRNAs may hence have other unpredictable side effects. Thirdly, a large number of miRNAs are only studied in animals. As we all know, mice and the human body have great differences. MiRNAs with functions in mice may not work in human body. Finally, miRNAs are also expressed in physiological state, so the determination of its therapeutic dose is also a difficulty. Therefore, further research may be needed to solve these problems.

### Potentiality of miRNAs in VMC Diagnosis

In VMC, miRNAs are differentially expressed not only in cells but also in blood. The stability of miRNAs in the blood can be maintained by binding RNA binding proteins and enveloping them within extracellular vesicles (Santos et al., 2020). There has been other disease using miRNAs as candidate diagnostic tool (Chen et al., 2019), but VMC has not yet been. Therefore, miRNAs in the blood may be used as a disease marker for VMC. Expression change of miRNAs in the circulation may help us assess the severity of VMC. Wang et al. evaluated the predictive value of miR-1 and miR-146b in VMC. MiR-1 decreased significantly in VMC, which was negatively correlated with the left ventricular shortening fraction and left ventricular ejection fraction. MiR-146b was significantly up-regulated in VMC, which was positively correlated with those two indexes (Wang D. et al., 2016). Therefore, miR-1 and miR-146b may be potential disease markers of VMC. Moreover, Corsten et al. Found that miR-208b and miR-499 were significantly increased in VMC, and correlated with blood troponin levels (Corsten et al., 2010). Furthermore, in a study on the correlation between miRNAs and child VMC pathology, the level of miRNA-208a increased significantly in the acute phase of the disease. Compared with the chronic phase, the level of miRNA-21 in the acute phase was also significantly higher (Goldberg et al., 2018). These studies indicated that miR-208a, miR-21, miR-1, miR-146b, miR-208b, and miR-499 will be promising biomarkers for VMC diagnosis. Compared with EMB, circulating miRNAs have the characteristics of less trauma, low cost, good sensitivity, and high specificity, so they may be used as potential disease markers for VMC differential diagnosis in the future. However, as a disease marker, miRNAs also meet some challenges. First of all, the quantitative methods, units of measurement and thresholds of miRNAs are different in different laboratories, which may limit their further use. Secondly, different methods of extracting miRNAs in different laboratories may produce contradictory results. Thirdly, the level of miRNAs has been fluctuating in different stage of VMC, so the point in time of obtaining miRNAs still needs further study.

## The Role of lncRNAs in VMC

LncRNAs belongs to non-coding RNAs with a length longer than 200 nucleotides (Li J. et al., 2018; Barangi et al., 2019; Chi et al., 2019). In many ways, lncRNAs are similar to mRNAs. However, compared with mRNAs, the transcription number and species conservation of lncRNA is low. The secondary structure of lncRNA, such as hairpin structure and stem ring structure formed by post-transcriptional modification, interacts with chromatin and protein to function. Based on the relative position to the protein-coding gene, lncRNAs can be divided into the following five categories: bidirectional lncRNA, the intron lncRNA, intergenic lncRNA, sense lncRNA, and antisense lncRNA. LncRNAs function mainly through chromatin remodeling, transcription and post-transcriptional regulation to influence gene expression (Atianand and Fitzgerald, 2014; Quinn and Chang, 2016; Dykes and Emanueli, 2017; Huang et al., 2018b). Previous studies have found that lncRNAs are involved in many pathophysiological processes, such as growth and development, immune, inflammation and tumor (Li Y. et al., 2018; Alessio et al., 2019; Chi et al., 2019; Wang et al., 2019; Zhang and Wang, 2019; Zhang K. et al., 2019).

Liu et al. confirmed that lncRNAs were differentially expressed in child acute fulminant myocarditis. Among them, 1645 lncRNAs were up-regulated and 1456 lncRNAs were down-regulated. Further bioinformatic analysis showed that T cell activation, T cell receptor complex, negative regulation of complement activation, immune response and T-helper 17 cell differentiation process may participate in the progress of VMC (Liu Q. et al., 2019). In addition, Zhang et al. recently found that lncRNA Ak085865 can promote the expression of anti-inflammatory M2 macrophages, reduce the number of proinflammatory M1 macrophages. LcRNA Ak085865 knockout mice were more susceptible to CVB3 induced VMC, suggesting that lncRNA Ak085865 may play a vital role in the pathogenesis of VMC by affecting macrophage polarization (Zhang et al., 2020). Furthermore, in isoproterenol-induced myocardial fibrosis model of VMC, lncRNA ROR promotes myocardial fibrosis by regulating the expression of C-Myc, suggesting that lncRNA act a vital factor in the chronicity of VMC (Zhang and Sun, 2019). From these existing studies, we can conclude that lncRNAs may be a potential therapeutic target by influencing macrophage polarization and myocardial fibrosis. However, there are few studies on lncRNAs in VMC, and more studies are needed to elucidate the role of lncRNAs.

## The Role of circRNAs in VMC

CircRNAs are non-coding RNAs widely expressed in eukaryotic cells, and different from the traditional linear RNAs because of they are circular molecules (Kristensen et al., 2019; Liu C. et al., 2019; Vo et al., 2019; Wilusz, 2019; Zlotorynski, 2019). The characteristics of circRNA are high abundance, structure stable and highly tissue-specific expression. They are mainly produced by the splicing of exon or intron sequences. Besides, reverse complementary sequences or RNA binding proteins (RBPs) are necessary for circRNAs production (Kristensen et al., 2019). Recent studies have shown that circRNAs act as a miRNA sponge to adsorb miRNA, protein sponge, protein scaffold and coding small peptide to complete their biological function (Xie et al., 2018; Zhang M. et al., 2018; Dong et al., 2019; Jiang and Ning, 2019; Liu J. et al., 2019; Lu et al., 2019). Many researchers have found that circRNAs play an important role in cardiovascular diseases such as myocardial infarction, heart failure, and atherosclerosis (Wang K. et al., 2016; Wang et al., 2017; Zhang F. et al., 2018). Zhang et al. confirmed that circRNAs were differentially expressed in fulminant myocarditis, and further bioinformatic analysis showed that many inflammatory or immune pathways, including TNF signaling pathway, Th1 and Th2 cell differentiation and T cell receptor signaling pathway, were involved in VMC (Zhang L. et al., 2019). Therefore, circRNAs may be a new therapeutic target for VMC by regulating the immune-inflammatory pathway. In addition, considering the stability of circRNAs, circulating circRNAs are also potential biomarkers for VMC. However, there is no detailed study on the mechanism of circRNAs affecting the development of VMC.

## Conclusions and Perspective

VMC is characterized as a localized or diffuse disease of myocardial parenchyma or interstitium caused by virus infection. The mechanism of VMC may be related to direct myocardial damage of virus infection and extensive damage of abnormal immune response after infection. Although large progress on VMC has been obtained in recent decades, the diagnosis, and treatment of viral myocarditis are still facing great challenges. With the discovery of more and more non-coding RNAs, non-coding RNAs have become the vital molecules of gene regulation in our cognitive range now. In the progress of VMC, non-coding RNAs regulate virus life cycle, immune and inflammatory response by targeting virus or host genes. By reviewing the mechanism of ncRNAs in CVB3-induced myocarditis, we summarized the non-coding RNA related potential disease markers and therapeutic targets of VMC. MiRNAs of VMC have been largely studied by researchers. However, the lncRNA and circRNA field of VMC is still virgin land to be developed. In the future, our researchers should pay more attention to the lncRNA and circRNA area.

## Author Contributions

ZY, CZ, YX, ZP, and LZ conceptualized the study. CZ and YX prepared the draft. CZ, YX, and ZY reviewed and edited the manuscript. CZ, YX, and ZL contributed to the visualization. ZY and HZ supervised the study. All authors contributed to the article and approved the submitted version.

## Conflict of Interest

The authors declare that the research was conducted in the absence of any commercial or financial relationships that could be construed as a potential conflict of interest.
